# Nogo Receptor Inhibition Enhances Functional Recovery following Lysolecithin-Induced Demyelination in Mouse Optic Chiasm

**DOI:** 10.1371/journal.pone.0106378

**Published:** 2014-09-03

**Authors:** Fereshteh Pourabdolhossein, Sabah Mozafari, Ghislaine Morvan-Dubois, Javad Mirnajafi-Zadeh, Alejandra Lopez-Juarez, Jacqueline Pierre-Simons, Barbara A. Demeneix, Mohammad Javan

**Affiliations:** 1 Department of Physiology, Faculty of Medical Sciences, Tarbiat Modares University, Tehran, Iran; 2 UMR CNRS 7221, Evolution des Régulations Endocriniennes, Département Régulations, Développement et Diversité Moléculaire, Muséum National d'Histoire Naturelle, Paris, France; 3 Department of Stem Cells and Developmental Biology at Cell Science Research Center, Royan Institute for Stem Cell Biology and Technology, ACECR, Tehran, Iran; Innsbruck Medical University, Austria

## Abstract

**Background:**

Inhibitory factors have been implicated in the failure of remyelination in demyelinating diseases. Myelin associated inhibitors act through a common receptor called Nogo receptor (NgR) that plays critical inhibitory roles in CNS plasticity. Here we investigated the effects of abrogating NgR inhibition in a non-immune model of focal demyelination in adult mouse optic chiasm.

**Methodology/Principal Findings:**

A focal area of demyelination was induced in adult mouse optic chiasm by microinjection of lysolecithin. To knock down *NgR* levels, siRNAs against NgR were intracerebroventricularly administered via a permanent cannula over 14 days, Functional changes were monitored by electrophysiological recording of latency of visual evoked potentials (VEPs). Histological analysis was carried out 3, 7 and 14 days post demyelination lesion. To assess the effect of NgR inhibition on precursor cell repopulation, BrdU was administered to the animals prior to the demyelination induction. Inhibition of NgR significantly restored VEPs responses following optic chiasm demyelination. These findings were confirmed histologically by myelin specific staining. siNgR application resulted in a smaller lesion size compared to control. NgR inhibition significantly increased the numbers of BrdU+/Olig2+ progenitor cells in the lesioned area and in the neurogenic zone of the third ventricle. These progenitor cells (Olig2+ or GFAP+) migrated away from this area as a function of time.

**Conclusions/Significance:**

Our results show that inhibition of NgR facilitate myelin repair in the demyelinated chiasm, with enhanced recruitment of proliferating cells to the lesion site. Thus, antagonizing NgR function could have therapeutic potential for demyelinating disorders such as Multiple Sclerosis.

## Introduction

Myelin associated inhibitory factors, including NogoA [1], myelin associated glycoprotein (MAG) [2] and oligodendrocyte myelin glycoprotein (OMgp) [3] are among the major factors known to inhibit regeneration in the CNS [4]. These factors bind to a common receptor called Nogo receptor 1 (NgR1) [5]. A large number of studies have shown that NgR is expressed by not only neurons [6] but also glial cells including oligodendrocyte progenitor cells (OPCs) [7], [8], astrocytes [9], microglia [10], macrophages [11], dendritic cells [12] and neural precursor cells [13]–[15]. It has been reported that NgR exerts multiple inhibitory effects in neural pathological conditions [16]–[18], including inhibition of neural precursor migration during CNS development [13]. While the focus of most of these studies has addressed the inhibitory roles of NgR or its ligands in axonal regeneration either in EAE demyelinating models [16], [19], [20] or non-demyelinating conditions [17], [21], [22], less is known about the roles of myelin inhibitory factors in demyelination condition in which the axons are intact or not targeted. Since it is well documented that myelin can protect axonal integrity and loss of myelin results in axonal loss and disability [23]–[26], it is important to better understand the role of myelin-derived inhibitory factors on myelin repair itself. This information is more pertinent given that NgR and its ligands are expressed in demyelinating lesions of MS tissues [9]. Chong et al. (2012) reported the role of NogoA in regulating oligodendrocyte myelination in vitro and in an in vivo focal model of demyelination [27]. The roles of other myelin-bound ligands of NgR, also likely involved in myelin regeneration, remained to be studied.

Here we targeted the common receptor (NgR) of myelin inhibitory factors to analyze its effects on myelin repair in an in vivo context of demyelination. We previously developed a focal model of demyelination in the optic chiasm of adult rats [28] and mice [29] and showed that remyelination could be followed functionally by assessing visual evoked potentials and structurally, by assessing demyelination extension [28]–[30]. Furthermore, we observed that the caudal part of the optic chiasm displayed more remyelination than the rostral part [30], probably due to its vicinity to the third ventricle, which is a known neurogenic region both in development [31] and adulthood [32]–[34]. In this study, we used the same focal model, targeting the caudal part of optic chiasm to investigate the effects of NgR inhibition during demyelination. We also examined the response of the neurogenic niche around the third ventricle during this process and followed remyelination by histological examination. Recording visual evoked potentials (VEPs) allowed us to evaluate the functional recovery of the optic chiasm. Our results demonstrate that siRNA directed against NgR significantly enhanced the remyelination process and functional recovery of optic chiasm. Further, NgR inhibition significantly increased the number of Olig2+ cells recruited in the lesion site and enhanced the numbers of third ventricle progenitor cells produced following chiasm demyelination.

## Material and Methods

### Animals

All animal studies were conducted according to the principles and procedures described in Guidelines for care and use of experimental animals and were approved by Tarbiat Modares University-Ethics Committee for Research on Animals. Eight week-old (25–30 gr) male C57BL/6 mice were purchased from Razi institute (Karaj, Iran) and JANVIER (Le Genest St Isle, France). Animals (five per cage) were kept under 12 h light/dark cycles with controlled temperature (22±20°C). Food and water were available ad libitum.

### Demyelination procedure

Animals were deeply anesthetized with intraperitoneal injection of Ketamine (100 mg/kg wt; Imalgen from Merial) and Xylazine (10 mgkg wt; Rompun from Bayer) diluted in 0.9% sterile saline. Meloxicam (Metacam from Boehringer Ingelheim) was used for analgesia throughout all operations.

Demyelination was induced by stereotaxic injection of 1 µl of 1% lysolecithin (LPC; sigma, St. Louis, USA) dissolved in 0.9% NaCl [28]. Mice were positioned in a stereotaxic device (Stoelting, USA) in a skull flat situation. The LPC was injected into the optic chiasm over 2 min, using the coordinates of 3.9 mm anterior to the Lambda, 5.75 mm deep from Dura surface and zero laterality [35]. The needle was kept in place for an additional 5 min to equilibrate tissue and inject solution, to avoid the possible reflux through the needle tract. Control animals were injected with equal volume of sterile saline.

### Interventions

For intracerebroventricular administration of siRNAs, animals were cannulated unilaterally in the right lateral ventricle (3.6 mm anterior to lambda, 1.1 mm lateral, and 2.2 mm deep from the Dura surface) [35]. siNgR was injected from cannula, 24 pmol (2 µl)/animal/days. Control groups received same volume of saline.

### Formulation of siRNA complexes

siRNA against pGL2 (for control group) and NgR1 (Rtn4r, 4 different sequences, Cat no: SI02722888, SI02699186, SI02748333, SI02678249) were purchased from Qiagen (validated siRNA). siRNA (24 pmol per animal) was diluted in glucose (5%) and mixed with monocationic lipid (IC10, Polyplus-transfection) at a ratio of 15 monocationic lipid nitrogens per RNA phosphate as described before [36], [37]. Complexes prepared at room temperature are stable for two hours after preparation. Unilateral injection of siRNA (2 µl) was performed every day from permanent cannula stereotaxically placed into right lateral ventricle. Details of siRNA sequences are presented in Table S1.

### Real time PCR

The rim of third ventricle of adult mice was dissected under a binocular microscope, snap-frozen in liquid nitrogen and stored at −80°C until processed. Total RNA was extracted based on the protocol provided with the RNAble reagent (Eurobio, Les Ulis, France). Concentration of total RNA was measured, and RNAs were stored in Tris 10 mM/EDTA 0.1 mM (*p*H 7.4) at −80°C. To quantify mRNAs 1 µg of total RNA was reverse-transcribed using High capacity cDNA Reverse Transcription kit (Applied Biosystems, Courtaboeuf, France). Control reactions without reverse transcriptase were done in parallel. Primers for the detection of NgR (Taqman gene expression assays, references: Mm00710554_m1) and control assay GAPDH (Mm99999915_g1) were purchased from Applied Biosystems. Direct detection of the PCR product was monitored by measuring the increase in fluorescence generated by the TaqMan probe (NgR, GAPDH) as described previously by Decherf et al. (2010) [38].

### Visual evoked potential (VEP)

Visual evoked potentials (VEPs) are evoked electrophysiological potential that can be extracted, using signal averaging, from the electroencephalographic activity recorded at the scalp. The VEPs can provide important diagnostic information about the functional integrity of the visual system. The surgical process is similar to the method described previously [28], [39], [40]. The animals were anesthetized and fixed to stereotaxic apparatus. To facilitate VEP recordings, a monopolar electrode was implanted into the occipital cortex (A: 0.0, L: ±3.0 mm, Lambda) and the anterior end of the skull served as the reference electrode. The electrodes were connected to a miniature receptacle, which was embedded in the skull with dental cement. For VEP recording, mice were immobilized in a box and allowed to adapt to darkness for 10 minutes. The cylinder was placed in a sound-attenuating dark and electrically shielded box (60 cm×60 cm ×60 cm). Flash light stimulation was delivered by a general evoked response stimulator (SMP-3100, Nihon Kohden, Tokyo, Japan) 300 times with a frequency of 0.5 Hz. The illumination of the reflecting surface was approximately 40 lx. Responses were amplified with high and low filter settings of 30 and 0.08 Hz, respectively, using a biophysical amplifier (AVB-10, Nihon Kohden), and displayed on a memory oscilloscope (VC-11, Nihon Kohden). Amplified waveforms were afterwards averaged (DAT-1100, Nihon Kohden). For each VEP recording, we studied the latency between the flashlight and the first negative or positive peaks.

### Cell tracing

Bromodeoxyuridine (5-bromo-2-deoxyuridine, (BrdU)) incorporates into DNA during S phase of the cell cycle; therefore it is appropriate for labeling of newly divided cells used for tracing of these cells while they are migrating. Mice received seven injections of Brdu (sigma, USA) at intervals of 2 hours (70 mg/kg each, i.p.) 24 hours prior to induction of demyelination. Using this tracing protocol, labeled cells is restricted to the germinative area of the CNS like lateral and third ventricles rims in controls. Afterward, detection of labeled cells in structures other than these areas implies that they originated from ventricular zone [41]–[44].

### Tissue processing

Mice were deeply anesthetized with Pentobarbital (130 mg/kg from Sanofi) on 3, 7, 14 days post lesion and perfused with a fresh phosphate buffered saline 0.1 M (PBS) and followed by 4% paraformaldehyde solution (PFA, pH 7.4). Brains were excised and post-fixed 2 hours in same fixative solution at room temperature and cryoprotected by 20% sucrose/PBS overnight then embedded in optimum cutting temperature compound, frozen and stored at −80°C. Coronal sections (12 µm) were obtained using a Cryostat (Leica, Rueil-mal maison, France) and collected on superfrost plus slides (Thermo Scientific). Coronal serial sections with 120 µm intervals (+20-880 µm) were analyzed. Therefore we were able to determine the migration pattern of third ventricle proliferative cells towards optic chiasm.

### Immunohistochemistry

Cryosections were rehydrated in 0.1 M PBS three times for 5 min (for myelin staining sections incubated in ethanol 95% for 10 min at RT washed two times with PBS) and incubated 1 h with blocking solution containing 10% normal goat serum (Vector Laboratories, France 501000) and BSA 2 mg/ml (Vector Laboratories- sp50-50) and 0.3% Triton ×100 (Sigma, France) in PBS. Slides were incubated overnight at 4°C with primary antibodies (Table S2) diluted in blocking solution and cover slipped. After slides were rinsed three times for10 min with PBS and incubated with secondary antibody (Table S3) for 1 h at RT. For double labeling with BrdU, sections were washed three times in PBS for 10 min and incubated in HCl 2N for 30 min at 37°C, after two washes 5 min in borate buffer (pH: 8.4) and PBS, sections were incubated with the same blocking solution and incubated overnight at 4°C with the anti Brdu antibody diluted in blocking solution. After washing, sections were incubated with secondary antibody for 1 h at RT. After several washes with PBS, brain sections were mounted in antifade reagent with DAPI (P3693, Invitrogen) and analyzed under fluorescent microscopy on an Olympus AX70 microscope and camera Olympus DP50.

For cell counting in the third ventricle and optic chiasm 12 µm sections were used. The number of total BrdU+ cells and Brdu+/Olig2+, Brdu+/GFAP+ or Brdu+/PSA-NCAM+ positive cells were averaged from three different levels 120 µm apart and three consecutive sections per level. For each brain (nine sections), data were expressed in number of cells per mm^2^ and are deduced from three mice per experimental group.

The extent of demyelination, as the ratio of lesion size per total area, was determined using Image J software [45]. Data obtained from individual animals in each group was averaged from 9 sections (n = 3 animals per treatment time point). The number of Iba-1+ cells in the lesion site were averaged from four sections per brain and data were expressed in number of cells per mm^2^ using a total of three mice per experimental group.

### Statistical Analysis

The result are expressed as mean± SEM. Data were analyzed by two way analysis of variance (ANOVA) followed by Bonferroni post-tests with Graph pad PRISM software (Graph pad software, Inc, San Diego. CA). Data were determined to be significant when p<0.05.

## Results

### siNgR successfully mediated knock-down of Nogo receptor in vivo

**Figure 1:**
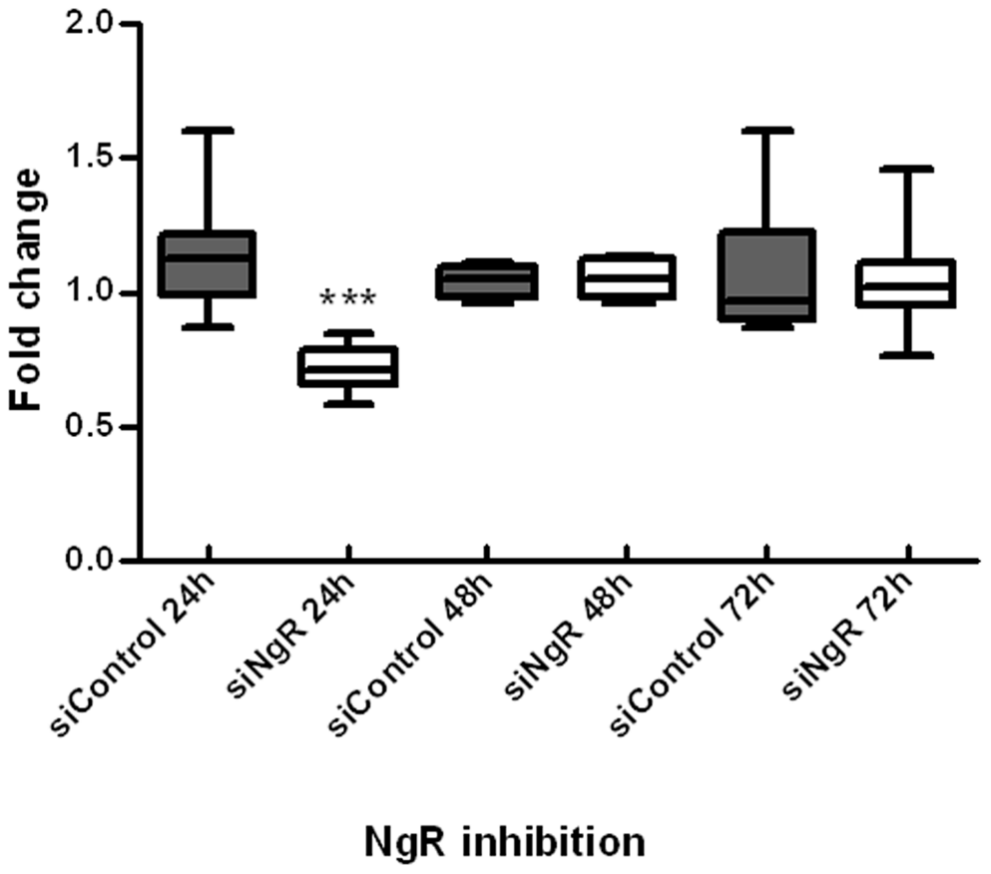
siRNA-mediated knockdown of *NgR*. qPCR of third ventricle samples show levels of *NgR* expression at 24, 48 and 72 hrs after single siRNA injection. Gene expression was normalized with *Gapdh*. Data are pooled from three independent experiments providing similar results (total number of mice for each data point  = 6). Graph shows fold changes in *NgR* mRNA using qPCR analysis. Boxes represent the fifth to ninety-fifth percentiles around the median with whiskers for minimum and maximum values. Statistical analysis used two-way ANOVA and Bonferroni's post -test. Treatment had significant effects on the level of NgR gene expression. NgR gene expression was decreased significantly 24 h after siNgR injection compared to siControl, the asterisk indicates that the difference between the pairs denoted are significant at the confidence levels ***p<0.001.

The putative role of NgR in myelin repair and NPCs migration was studied by a loss of function strategy based on RNA interference. Remaud (et al. 2013), showed that siRNA/IC10 is the first generation of lipid based siRNA vectors to provide efficient, spatially and temporally defined knockdown in the adult brain [37]. The observed distribution of labled siRNA indicated that IC10 vectorizes siRNA in to cells expressing markers characteristic of NSC and transient amplifying progenitor cells (TAPs) in the adult SVZ [37]. To knock down NgR, a validated siRNA targeting NgR1 complexed with IC10 was injected stereotaxically into the lateral ventricle of adult mice brain. The areas lining the third ventricle of brain injected with either NgR (siNgR) or Control (siControl) were carefully sampled under dissecting microscope 24, 48 and 72 hrs after siRNA injection. RNA was extracted for qPCR analysis. A significant (p<0.001) decrease (0.40 fold change) in NgR mRNA expression was observed 24 hrs after the injection, in the group injected with NgR-siRNA when compared to control-siRNA (Fig. 1). No significant differences were observed between siNgR and siControl groups for later time points. This result indicates that siRNA-mediated knockdown of NgR was efficient and transient. To knockdown NgR during the process of the LPC demyelination/remyelination (2 weeks), 24 pmol siNgR/IC10 were daily injected in the ventricle of stereotaxically cannulated adult mice.

### Functional recovery in demyelinated optic chiasm of mice following NgR inhibition

**Figure 2:**
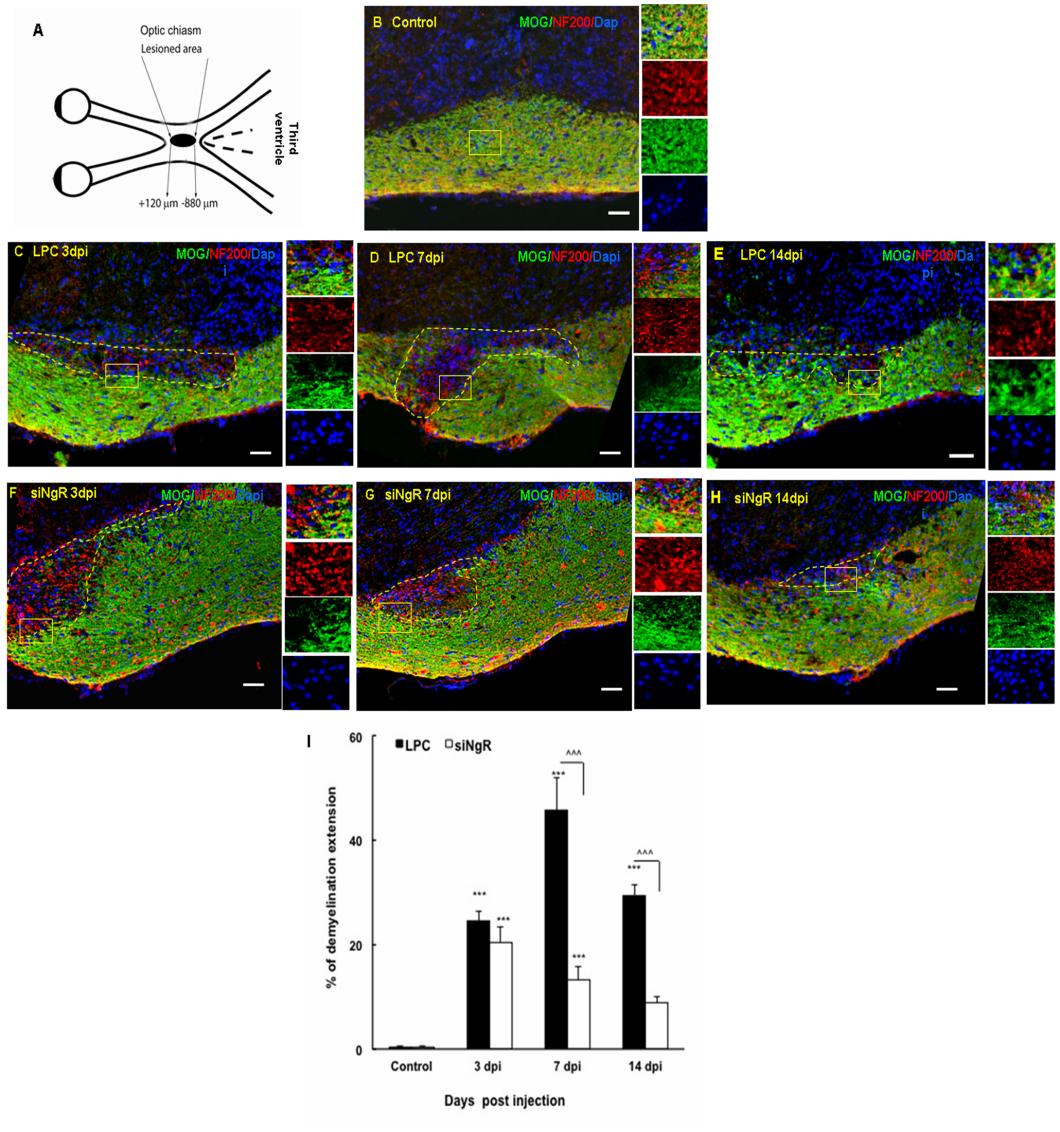
Extent of demyelination is decreased following NgR knock down. Coronal sections of adult mouse brains (12 µm) treated with saline, LPC and LPC+siNgR were double stained with MOG (green) and NF200 (red) and nuclei are labeled with DAPI (blue). (A) Schematic picture of injection site and demyelination area. (B) Saline-treated chiasm at 7 days post injection (dpi), no detectable demyelination is seen 7 days after a single injection of saline (Control). (C–E) Demyelination at optic chiasm of LPC-treated animals at 3, 7 and 14 dpi, respectively. (F–H) Demyelination at optic chiasm of LPC+siNgR treated animals at 3, 7 and 14 dpi, respectively. (I) The extent of demyelination in different groups are quantitatively analyzed and presented as percent of total area. Control group represents demyelination level in animals treated with saline at dpi 7. Statistical analysis used two-way ANOVA and Bonferroni's post -test. Treatment and time had a significant effect on the remyelination process. In LPC treated-animals significant demyelination is seen at 3, 7 and 14 dpi compared to saline (^***^P<0.001). At 14 dpi, demyelination was partially reduced compared to 7 dpi. Between groups, there was a significant reduction of demyelination in LPC+siNgR 7 dpi compared to LPC 7 dpi (^∧∧∧^P<0.001) and in LPC+siNgR 14 dpi compared to LPC 14 dpi (^∧∧∧^P<0.001). Each data point shows data obtained from experiments carried out on three mice (n = 3), and represents Mean ± SEMs, Bars: 50 µm, Dashed line indicates lesion area.

To investigate the role of NgR in a demyelinating context, we induced a focal lesion in the optic chiasm using LPC injection. We confirmed the physiological pertinence of the model by immunostaining against the myelin specific marker MOG (Fig. 2A) and LFB staining (data not shown). We observed that, at all time points axons were not affected by LPC injection when tissues were stained for axonal neurofilament (Fig. 2C–H). No demyelination was detected in the optic chiasm of saline-treated controls (Fig. 2B). Demyelination was observed as early as 3 days post injection. Demyelination extent was assessed as percentage of total area of optic chiasm. The demyelination extent was maximal at 7 dpi compared to controls (p<0.001, Figs. 2C, D). In LPC treated animals after 14 days the extent of demyelination was reduced compared to LPC 7 dpi (Fig. 2E). In siNgR treated animals the demyelination area was not different at 3 dpi compared to LPC but was considerably reduced at day 7 and 14 post injection (p<0.001 for all comparisons, Figs. 2F–H).

**Figure 3:**
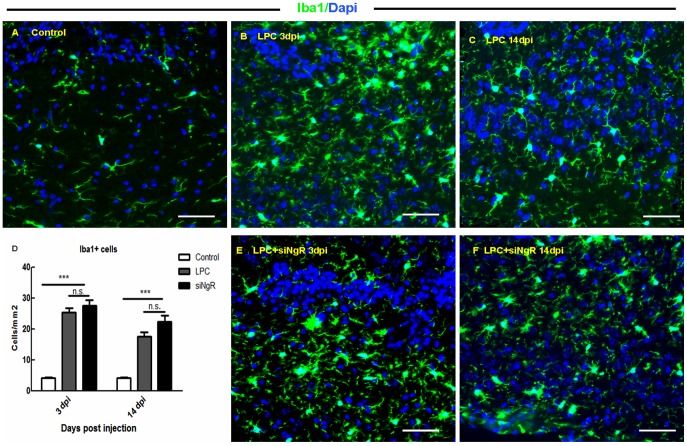
Macrophages and microglia response to LPC-induced demyelination. Sections from lesion sites were stained for Iba-1 (green) and DAPI (blue) at 3 and 14 dpi in both LPC and LPC+siNgR treated groups. (A) The number of Iba-1+ cells in saline treated animals (Control) was low at 3 dpi. (B–C) Increased number of microglia and macrophages in the lesion site following LPC induced demyelination at 3 (B) and 14 dpi (C). (E–F) In siNgR treated mice in LPC induced lesion also the number of Iba-1+ cells was increased at 3 (E) and 14 dpi (F). (D) Iba1+ cells per area/mm^2^ are averaged from the counts of four sections of each chiasm (n = 3). Statistical analysis used two-way ANOVA with Bonferroni's post-test. The effects of treatment and time were significant. Further analysis using post-test showed significant difference between Control and LPC treated mice (p<0.001), and between Control and LPC+siNgR treated mice (p<0.001) at 3 and 14 dpi. The difference between the LPC and LPC+siNgR treated groups was non-significant (n.s.). Data are expressed as Mean ±SEMs, Bars: 50 µm.

Since NgR is expressed also by microglia and macrophages [10], [12]; and these cells are involved in LPC-mediated lesions and/or remyelination [46], [47], we carried out immunocytochemistry on sections to label Iba-1+ cells at the lesion site and counted the number of Iba-1+ cells in LPC and LPC+siNgR treated animals at 3 and 14 dpi and compared it to saline treated chiasm evaluated at dpi 3. The number of Iba-1+ cells significantly increased in LPC and LPC+siNgR treated groups at all time points compared to control (p<0.001, Fig. 3 A–F). In LPC+siNgR groups the number of Iba-1+ cells was slightly higher than LPC but these changes were non-statistically significant (Fig. 3D).

**Figure 4:**
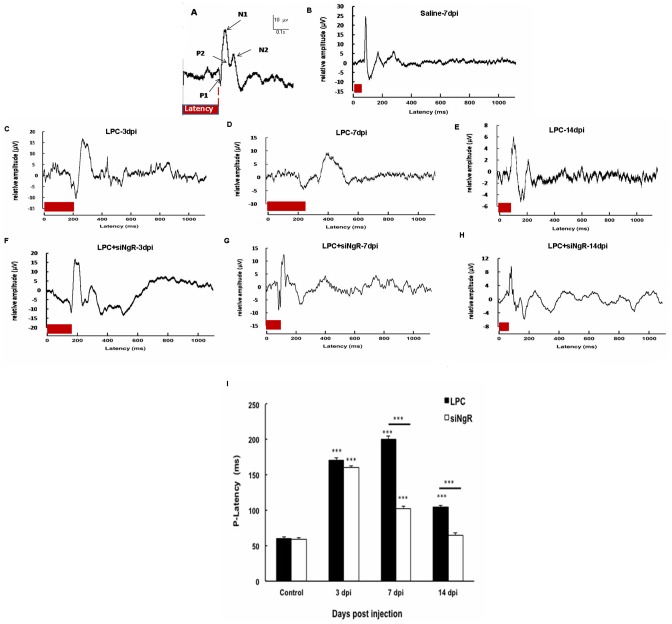
Functional recovery is induced by NgR inhibition in demyelinated optic chiasm. (A) Visual evoked potential (VEP) sample recordings from electroencephalographic activity and its components. The trace represented is the average of 300 sweeps of 300 ms duration with 1 Hz frequency. P1 latency (red bar) was measured by Biochart software. (B) VEP sample from animals treated with saline inside the chiasm (control) recorded at 7 dpi. (C–E) Changes in the P1 wave latency at 3, 7 and 14 dpi in the LPC treated animals. (F–H) VEP sample recordings from LPC+siNgR treated animals at 3, 7 and 14 dpi. (I) Quantitative analysis of changes in P1 latency in different groups. Statistical analysis used two-way ANOVA with Bonferroni's post-test. Treatment and time had a significant effect in this study. In LPC treated animals P1- latency was increased at 3, 7 and 14 dpi compared to control (all, ^***^p<0.001) but was partially diminished at 14 dpi (E). In LPC+siNgR group, there was a significant increase in p-latency at 3 and 7 dpi compared to control (both, ^***^P<0.001). NgR inhibition induces functional recovery at 7 and 14 dpi compared to LPC 7 and LPC 14, respectively (both, P<0.001) and there was no significant change in p-latency between LPC+siNgR and Control at14 dpi. Data was pooled from three independent experiments on mice (n = 6), Bars: Mean ± SEMs.

To functionally investigate the effect of NgR knockdown in demyelinated optic chiasm, Visual Evoked Potentials (VEPs) were recorded from the occipital cortex of the control and treated mice. VEPs consist of specific P1–N1, N1–P2 and P2–N2 components (Fig. 4A). P1-N1 component represented the more stable wave and was therefore used for measuring timing of the response to the light stimulus [28], [48]. P1 latency was calculated from VEP profiles as shown in (Fig. 4A). The reference is given by the mean P1 latency observed in the saline group (58 ms) (Fig. 4B). LPC treatment significantly increased the P1 latency observed at 3, 7 and 14 dpi (n = 6, p<0.001). However, the P1 wave latency was reduced at 14 dpi compared to 7 dpi in LPC group (Figs. 4C–E). Treatment with siNgR reduced the P1 latency at 7 and 14 dpi compared to LPC 7 and 14 dpi (p<0.001, Figs. 4C–I). These electrophysiological results were consistent with our histological data (Figs. 2C–E) showing the reduction of extent of demyelination at 14 dpi. Demyelination was observed in all treatment groups but siNgR treated mice presented reduced demyelination. This difference was particularly remarkable at 7 and 14 dpi (Fig. 2, 4). The reduction in the P1 latency and demyelination extent demonstrated that knockdown of NgR potentiates repair process of demyelinated optic chiasm by inducing a functional recovery. Next, to assess the cellular basis of this functional recovery we tracked the production and migration of progenitor cells to the demyelinated chiasm following NgR inhibition.

### Characterization of BrdU+ cells in demyelinated optic chiasm

**Figure 5:**
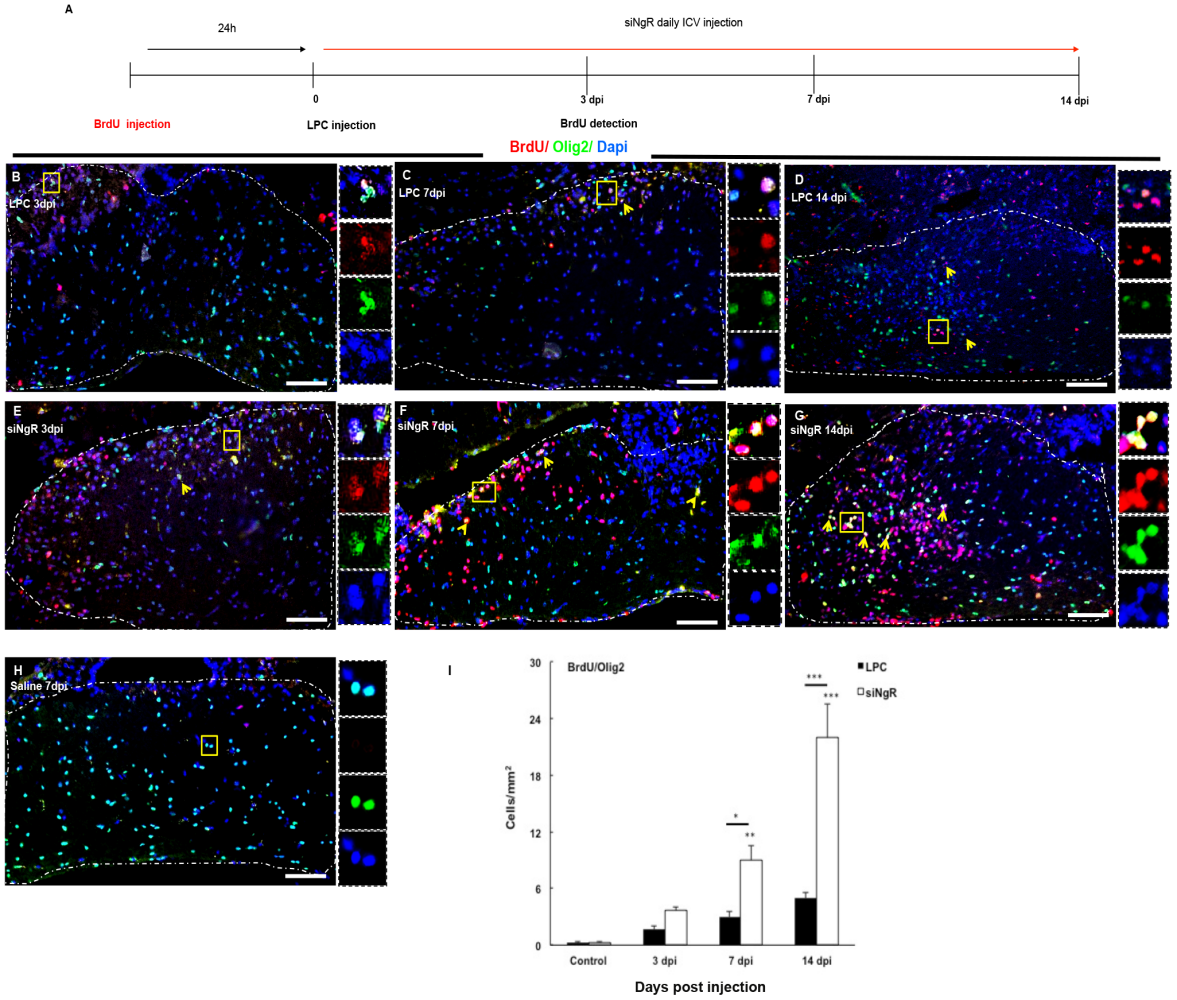
Increased BrdU+/Olig2+ cell numbers in demyelinated optic chiasm following NgR inhibition. Double immunohistochemistry (IHC) of BrdU (red) and Olig2 (green)-labeled cells was done on coronal section of optic chiasm in different groups. (A) Time line of BrdU injection and sampling in different groups. Mice received seven injections of Brdu at intervals of 2 hrs, 24 hrs prior to demyelination, and were sacrificed 3, 7 or 14 days after LPC injection. (B–D) BrdU+/Olig2+ cells in optic chiasm of LPC-treated animals at 3 (B), 7 (C) and 14 (D) dpi. (E–G) Optic chiasm of LPC+siNgR treated animals at 3 (E), 7 (F) and 14 (G) dpi. (H) Low number of BrdU+/Olig2+ cells in optic chiasm of saline-treated animals at 7 dpi (Control). Arrows indicate double-labeled cells and Square shows the cells magnified in inset. Dashed line indicates optic chiasm border. (I) BrdU+/Olig2+ cells per area/mm^2^ are averaged from the counts of nine sections of each chiasm. Statistical analysis used two-way ANOVA with Bonferroni's post-test. The effects of treatment and time were significant (p<0.001). In LPC treated groups the number of BrdU+/Olig2+cells was increased over the time but changes were not significant compared to Control. In LPC+siNgR treated animals, the number of BrdU+/Olig2+cells at 7 and 14 dpi in the lesion site was increased compared to control (^**^p<0.01, ^***^p<0.001; respectively). Between groups significant changes exist at 7 dpi (^*^p<0.05) and 14 dpi (^***^p<0.001). Data are expressed as Mean±SEMs, n = 3, Bars: 100 µm.

**Figure 6:**
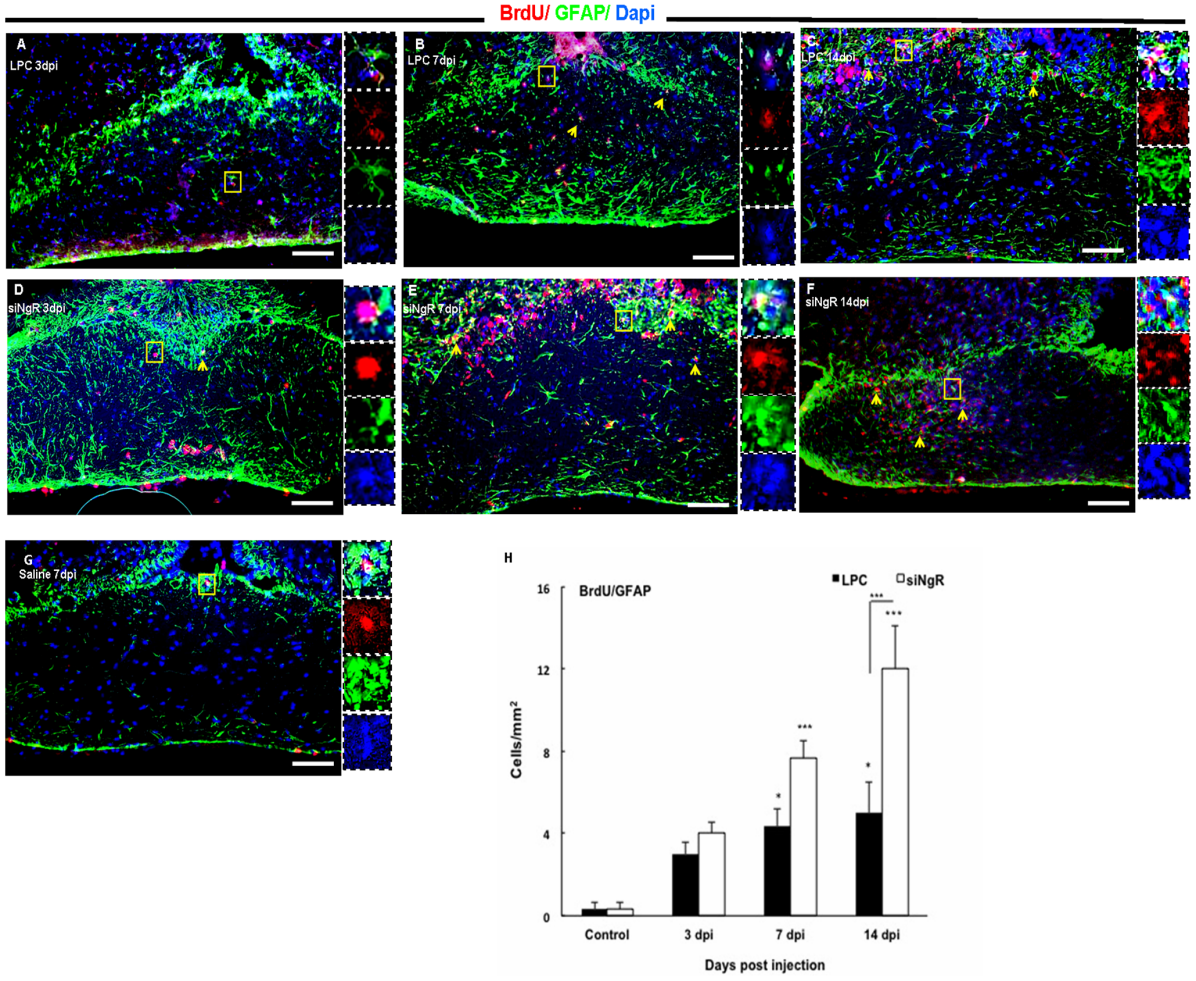
Increased numbers of BrdU+/GFAP+ cells within the lesion site following NgR inhibition. (A–C) Immunofluorescent images of Optic chiasm in LPC-treated animals at 3, 7 and 14 dpi. The number of BrdU+/GFAP+ cells at LPC 3 dpi (A), LPC 7 dpi (B) and 14 dpi (C) was increased. (D–F) Optic chiasm images of LPC+siNgR treated animals at 3 (D), 7 (E) and 14 dpi (F). The number of BrdU+/GFAP+ cells at 7 and 14 dpi in the lesion site was increased. (G) The number of BrdU+/GFAP+ cells in optic chiasm of saline-treated animals (Control) at 7 dpi was low. Arrows indicate double-labeled cells and Square shows the cells magnified in inset. (H) BrdU+/GFAP+ cells per area/mm^2^ are quantified and averaged from the counts of nine sections of each chiasm. Statistical analysis of the differences between numbers of BrdU+/GFAP+ cells in the optic chiasm of all groups was done by two-way ANOVA followed by Bonferroni's post-test. Differences between groups were significant (p<0.001). Post-test showed that the number of BrdU+/GFAP+ cells in LPC treated animals at 7 dpi and 14 dpi was increased significantly compared to Control (both, p<0.05). In LPC+siNgR treated animals the number of BrdU+/GFAP+ cells at 7 and 14 dpi in the lesion site was increased significantly compared to Control (both, ^***^p<0.001). NgR inhibition enhance the number of BrdU+/GFAP+ cells in optic chiasm compered to LPC groups and this change was significant between siNgR 14 dpi and LPC 14 dpi (^***^p<0.001). Data are expressed as Mean ± SEMs, N = 3, Bars: 100 µm.

To study the effect of siNgR on cell repopulation within the lesioned area, cells were labeled with BrdU before demyelination induction and were analyzed at 3, 7 and 14 dpi. The analysis showed that in saline treated animals few BrdU+ cells were present (Fig. 5H, 6G). However, the total number of BrdU+ cells increased progressively in LPC treated animals and at 14 dpi this increase was significant compared to controls (p<0.001, Table 1). In siNgR treated animals the number of total BrdU+ cells showed an almost 2 fold increase compared to LPC groups (p<0.001, Table 1). To characterize the BrdU+ labeled cells, tissues were stained for Olig2, GFAP, PSA-NCAM. The number of BrdU+/Olig2+ cells in the optic chiasm of LPC group did not change significantly over the time, however, the number these cells increased markedly in optic chiasm of siNgR-treated animals from 7 to 14 dpi as compared to controls (p<0.01, p<0.001, Fig. 5B-H). This increase was also significant between LPC 7, 14 dpi and siNgR 7 and 14 dpi (p<0.05, p<0.001, Fig. 5 I). Numbers of BrdU+/GFAP+ cells were significantly increased in LPC 7 and 14 dpi compared to control (p<0.05, Fig. 6 A–C). However, in siNgR treated LPC-groups the numbers of these cells significantly increased at 7 and 14 dpi compared to controls and LPC 14 dpi respectively (p<0.001, Fig. 6 A–H). BrdU+ cells in the optic chiasm were principally co-labeled with Olig2 and GFAP, but none, in any experimental group, expressed PSA-NCAM.

**Table 1:** Total number of BrdU+ cells in third ventricle and optic chiasm in different groups.

Total number of BrdU+ cells	Saline 7 dpi	LPC 3 dpi	LPC 7 dpi	LPC 14 dpi	siNgR 3 dpi	siNgR 7 dpi	siNgR 14 dpi
Third ventricle	0.3±0.12	7.3±1.97	9.1±1.5*	6±1.16	21.3±2.5***	16±2.49**	7±1.55∧∧
Optic chiasm	3.66±0.88	10±1.73	15±1.82	25.3±3.6***	14.67±2.6	32±2.25***	50.6±3.7

Data in all groups evaluated by one way ANOVA and *p<0.05, **p<0.01 and ***p<0.001 show significance changes compared to saline and ^∧∧^p<0.01 compared to siNgR 7 dpi.

### Third ventricle progenitor cells activation to demyelinated optic chiasm is increased in response to NgR knockdown

We previously showed that LPC-induced demyelination in adult rat optic chiasm activated proliferation in the subventricular zone (SVZ) around the third ventricle [30]. In this study we observed that inhibition of NgR resulted in a greater response from third ventricle following LPC lesion in the mouse optic chiasm. The analysis showed that progenitor cells residing in third ventricle wall were not mitotically active in saline treated animals (Fig. S1A). Third ventricle SVZ was reactivated in response to LPC induced lesion in optic chiasm. We observed that total number of BrdU+ cells increased at day 3 and 7 dpi in LPC treated mice. However in siNgR+LPC treated animals the number of total BrdU+ cells showed a two-fold increase compared to LPC groups at 3 and 7 dpi. The total number of BrdU+ cells at 14 dpi decreased significantly compared to siNgR 7 dpi (p<0.001, Table 1).

**Figure 7:**
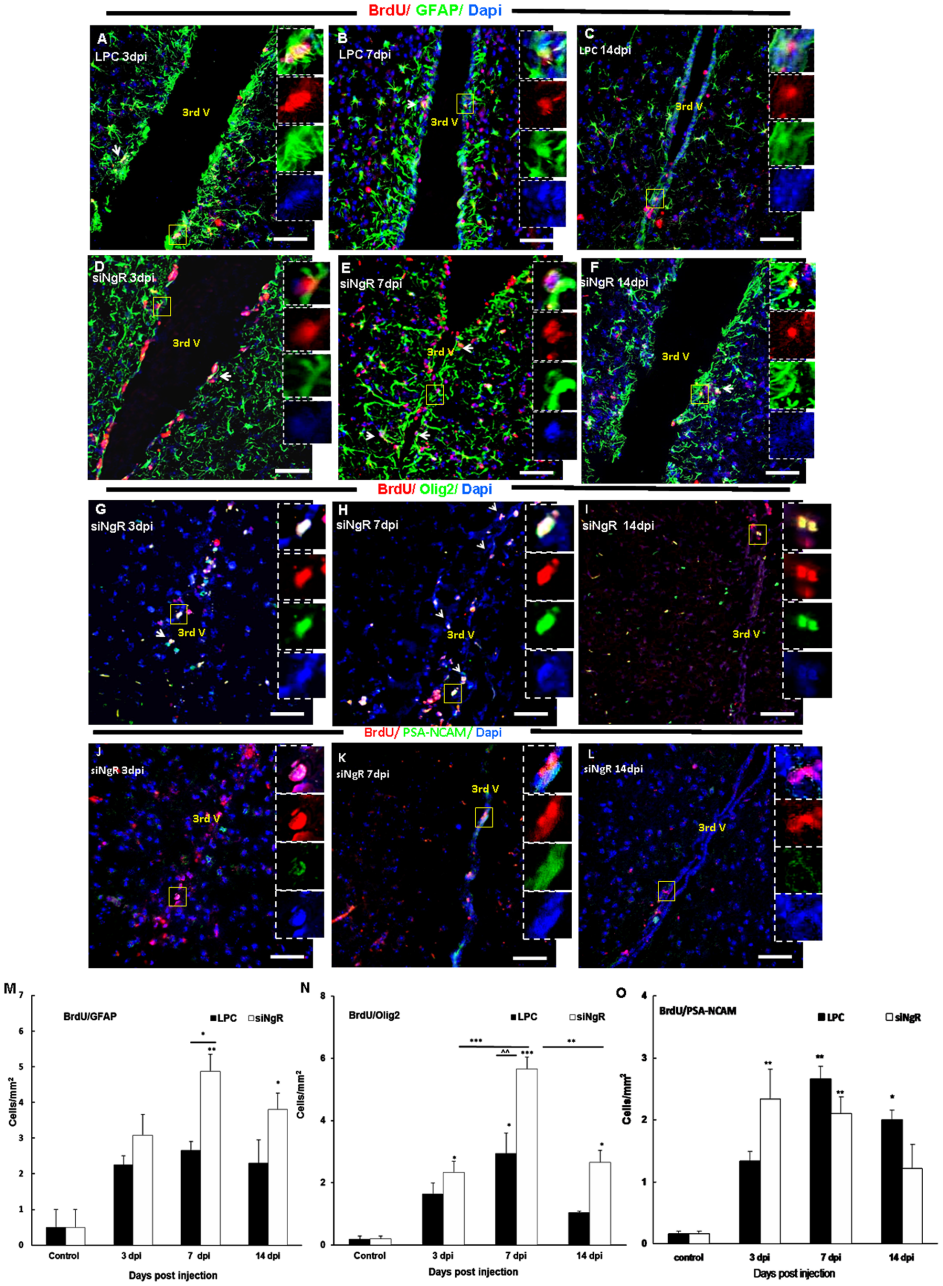
Third ventricle progenitor cell activation in response to LPC-induced lesions in optic chiasm is greater following NgR inhibition. Double labeling of BrdU (red) with Olig2, GFAP or PSA-NCAM (green) was done in coronal sections of third ventricle area in different groups. (A–C) BrdU+/GFAP+ cells in the rims of third ventricle of LPC group at 3 (A), 7 (B) and 14 dpi (C). (D–F) BrdU+/GFAP+ cells in the rims of third ventricle of LPC+siNgR treated animals at 3 (D), 7 (E) and 14 dpi (F). (G–I) BrdU/Olig2 double staining in third ventricle of LPC+siNgR treated animals at 3 (G), 7 (H) and 14 (I) dpi (see fig. S1D–F for BrdU+/Olig2+ cells in LPC group). (J–L) BrdU/PSA-NCAM positive cells in the third ventricle of LPC+siNgR treated animals at 3 (J), 7 (K) and 14 dpi (L) (see fig. S1G-I for BrdU+/PSA-NCAM+ cells in LPC group). Square shows the cells magnified in inset. Arrows show double marker positive cells. (M–O) Histograms show quantification of double marker positive cells, BrdU+/GAFP+ (M), BrdU+/Olig2+ (N) and BrdU+/PSA-NCAM+ (O) in different groups. Double marker positive cells per area/mm^2^ are averaged from the counts of nine sections of each brain, n = 3. Statistical analysis used two-way ANOVA with Bonferroni's post-test. Differences between groups were significant (p<0.001). The number of BrdU+/GFAP+ cells in third ventricle of LPC 3, 7 and 14 dpi was increased but changes were not significant compared to Control 7dpi (mice received saline inside chiasm), but this type of cells in LPC+siNgR treated animals at 3, 7 and 14 dpi were considerably increased compared to Control (^*^p<0.05, ^***^p<0.001, ^**^p<0.01, respectively) (M). The number of BrdU+/Olig2+ cells was considerably increased in siNgR-LPC group at 3, 7 and 14 dpi compared to Control (^**^p<0.01, ^***^p<0.001; respectively) (N). Additionally in these animals, at 7 dpi the number of BrdU+/Olig2+ was considerably increased compared to LPC 7 dpi (^∧∧∧^p<0.001) (N). The number of BrdU/PSA-NCAM positive cells in the third ventricle of LPC+siNgR treated animals was significantly increased at 3 and 7 dpi (both, p<0.01), while it was significantly increases in LPC treated animals at 7 and 14 dpi (p<0.05, p<0.01; respectively). Data are expressed as Mean ±SEM, Bars: 50 µm.

To identify proliferating cell types in third ventricle, BrdU+ cells were co-labeled with the antibodies against Olig2, PSA-NCAM and GFAP. Numbers of BrdU+/GFAP+ cells were not significantly modified in LPC treated animals compared to controls, whereas in siNgR treated animals BrdU+/GFAP+ cells significantly increased in the third ventricle SVZ at 3, 7 and 14 dpi compared to controls (p<0.05, p<0.001, p<0.01, Fig. 7A–F, M) respectively. Numbers of BrdU+/Olig2+ cells in LPC groups increased at 3 and 7 dpi compared to control (p<0.05, p<0.001 respectively), then decreased slightly at 14 dpi, (Fig. S1A–D, N). In siNgR-treated animals, BrdU+/Olig2+ cell numbers increased at 3, 7 and 14 dpi compared to controls (p<0.01, p<0.001, p<0.001, respectively) with an almost two fold increase in siNgR 7 dpi compared to LPC 7 dpi (P<0.001, Fig. 7 G–I, N). The number of these cells then decreased at siNgR14 dpi compared to siNgR 7 dpi (Fig. 7 H–I, N).

In LPC alone and siNgR-treated animals, some third ventricle BrdU+ cells expressed PSA-NCAM; and the number of BrdU+/PSA-NCAM+ cells in these groups was significantly higher than control group, however, these changes were not statistically significant when LPC and LPC+siNgR groups were compared (Fig. 7 J–L, O; Fig S2C, G–I).

Taken together, our results suggest that dividing cells are mobilized toward the lesion area in demyelinated optic chiasm. These cells mainly expressed Olig2 and GFAP, indicating a glia fate for progenitor cells at this neurogenic region.

## Discussion

It has been reported that NgR is expressed not only by neurons [6] but also by other neural cells including neural stem cells [13]–[15], oligodendrocyte precursor cells [7], [8], astrocytes [9], Schwann cells [12], microglia [10] or even non-neural cells [49]. This suggests a widespread role for NgR in many biological systems. NgR and its ligands have shown to hamper plasticity following neural damage or in CNS diseases [16]–[20]. Interacting with NgR, myelin derived inhibitory factors are the major candidates affecting axon outgrowth in the adult CNS [5]. Numerous experimental approaches have been used to assess the role of these inhibitory factors or their common receptor on axonal repair, including molecular blocking strategies, transgenic mice and models of CNS damage from spinal cord injury [17], [18], stroke [22] to immune-mediated models of demyelination [16], [19], [20], [50]. It is well documented that inhibition of NgR can functionally enhance axonal repair, but divergent neurobiological outcomes have been observed following NgR inhibition in EAE models. Since OPCs, NPCs and also immune cells express NgR, the effect of NgR inhibition in non-immune models of demyelination has not been fully investigated.

Here, by partially blocking NgR, we provide functional evidence from an inhibitory role of NgR on progenitor cell repopulation and myelin repair following optic chiasm demyelination. Recording visual evoked potentials following NgR knocking down enabled us to assess the myelin repair process electrophysiologically.

To locally inhibit NgR, we injected siRNA against NgR into lateral ventricle of adult mouse and evaluated the efficiency of NgR blockade by real time PCR for NgR mRNA in the tissue isolated from third ventricle surroundings which is anatomically close to the optic chiasm. Real time PCR quantification showed that the siRNA inhibits NgR gene expression by up to 40% 24 hours after in vivo injection. We thus applied siRNAs against NgR (siNgR) on a daily basis through permanent cannula. Our previous study [37] showed that with this technique the in vivo viability of siRNA is about 24 hours. This short term knock-down efficiency actually ensures that such RNAs interference method exerts transient and hence reversible effects [37].

This siNgR was applied following LPC-induced demyelination localized in the optic chiasm. Our results show that in animals treated with LPC alone, lesions were maximal at 7 dpi. In contrast, in groups receiving siNgR over 14 days LPC-induced demyelination was maximal at 3 dpi and progressively and significantly reduced over the following 10 days (7 to 14 dpi). Our histological findings were functionally substantiated with VEPs recording. We used P1-latency delay, which closely reflects the degree of demyelination in the visual pathway. Recent studies show that latency prolongation of VEPs corresponds to size of the lesion area in the visual pathway in optic nerve demyelination or neuritis [28], [48], [51]. Here we show that LPC injection into the optic chiasm leads to increased P1-latency at 3 and 7 dpi, followed by significant reduction in the latency at 14 dpi. However, P1-latency was recovered as early as 7 days post lesion in siNgR treated groups. Thus, inhibition of NgR facilitates functional recovery of visual pathways. This data fits with that of Chong et al. [27] who showed that mice lacking NogoA (NogoA-/-) have increased myelinogenic potential of oligodendrocytes, enabling enhanced remyelination after LPC-induced demyelination in the adult spinal cord [52]. The inhibitory effect of NogoA on OPCs differentiation in vitro has been also show by Syed and others (2008) [15]. However, Wang and others reported that exposure of neural stem cells with nogo-66 promotes glial but inhibits neuronal differentiation in vitro [16].

Furthermore, the time course of repair with siNgR in our data fits with the recent report by Petratos and others [16] in which they showed a reduction in clinical score, inflammatory cell infiltrates, demyelination and axonal degeneration in EAE-induced *NgR-/-* mice. More specifically, their data shows that myelin repair in EAE-induced *NgR-/-* mice occur between 12-18 dpi [19], [53]. Further, vaccination against Nogo A [20], or its systemic silencing using intravenously injected siRNA for NogoA suppresses EAE [50]. However, Steinbach and others reported that complete depletion of NgR did not promote functional recovery in another EAE model [10], [49]. These different observations in EAE models are most likely due to the expression of NgR by immune cells as well as neural cells which exert inhibitory roles on their migration [11]. We used immunocytochemistry with an Iba-1 antibody on sections and observed that the number of Iba-1+ cells in LPC and LPC+siNgR groups were significantly increased at 3 and 14 dpi. The number of Iba-1+ cells was slightly increased in the lesion site when NgR was partially inhibited; however, the result was not statistically significant compared to LPC-treated animals. So, we suggest that partial blockade of NgR in our study might not favor the myelination through inflammatory cells mediated mechanisms. Although it has been reported that NgR expression by dendritic cells [10], microglia [12] and macrophages [46] helps repulsion of these cells from myelin debris or the lesion site, Kotter et al. (2001) showed that depletion of these cells in LPC-induced demyelination by inhibiting inflammatory responses impairs remyelination [47]. Furthermore, Miron et al. (2013) recently reported that anti-inflammatory microglia and macrophages drive oligodendrocyte differentiation during CNS remyelination [7]. So, we suggest that partial blockade of NgR in our study might not favor the repair through inflammatory cells mediated mechanisms.

It has been reported that myelin associated inhibitory factors inhibit OPCs migration [13] and depletion of NogoA increases progenitor cell migration during cortical development [28], [30]. To investigate the effect of NgR inhibition on precursor cell recruitment in vivo, we pre-labeled the proliferating cells by BrdU before induction of LPC lesion in adult mouse optic chiasm. Our previous data showed that BrdU+ cells were recruited to the demyelinated rat optic chiasm over time and this was concurrent with recovery in myelin repair and VEP features [54]. In the current study we adjusted this model in mice to assess the effect of NgR inhibition on precursor cell repopulation. Interestingly our results reveal that total number of BrdU+ cells was significantly increased in demyelinated mice treated with siNgR over time when compared to LPC-induced control mice. Furthermore, we observed that these recruited cells expressed Olig2 but also GFAP markers of glial cells. Our observations show that NgR inhibition functionally increases myelin repair, as evidenced by an enhancement in progenitor cell migration to the lesion site. The mechanisms by which NgR inhibition favors cell recruitment need to be addressed. However, since there is evidence that OPC migration is governed by PDGF-AA and FGF-2 [55], and as FGF-2 has been suggested to interact with NgR1, which antagonizes FGF-2 signaling [13], it is plausible that NgR expressed on oligodendroglial lineage cells may play a role in their migration. Also NgR blockade in vitro increases the total covered distance and the maximum speed of migration of precursors in neurospheres [56]. Nogo A could also enhance the adhesion and inhibit the migration of OECs via NgR regulation of RhoA [10], [12]. Roles for NgR on immune cell migration [57] or human glioma cells [32], [34] have been also reported.

In adult human and rodents, the optic chiasm is anatomically proximal to the third ventricle. Several studies showed that third ventricle neurogenic zone contains multipotent cells that can give rise into neurons, oligodendrocytes and astrocytes in vitro [30]. Xu and others (2005) reported that cells located in ependymal layer of the third ventricle were able to migrate into hypothalamic parenchymal regions and differentiate into functional neurons. Our previous study [41] also showed that progenitor cells in the third ventricle surroundings could be reactivated by adjacent chiasm demyelination. Ernest and others (2005) also showed that in response to brain damage, there was a significant increase in numbers of BrdU+ cells in third ventricle. We also observed that numbers of BrdU+ cells were significantly greater than control at 7 dpi when NgR was inhibited. Characterization of cell types revealed that NgR inhibition plus chiasm demyelination increases BrdU+/Olig2+ or BrdU+/GFAP+ populations in comparison with control animals.

The number of Olig2+ precursor cells significantly decreased in third ventricle in NgR treated mice at 14 dpi. The reduction of BrdU+/Olig2+ cells in the third ventricle at days 7 to 14 was correlated with the appearance of increased numbers of these cells within the chiasm as early as 7 dpi. These increases at 14 dpi suggest that progenitor cells located in third ventricle were mobilized and migrated in response to the LPC-induced demyelination in the optic chiasm. These mobilization and migration process were more marked when NgR was blocked. Mobilization of proliferating cells from SVZ of lateral ventricle in response to periventricular demyelination has been also reported [30]. We also observed that the number of BrdU+/GFAP+ cells increased in the area around the third ventricles in siNgR treated animals. GFAP reactivation may be an immediate astrocytic response to damage as in other brain areas. GFAP gene expression increases in response to demyelination in optic chiasm [58] indicating astrocyte reactivity in the lesion site. Although astrocyte activation has positive and negative effects on survival, migration and differentiation of OPCs, the data from GFAP-/- mice suggest that astrocytes are required for the long-term maintenance of CNS remyelination [59] and OPC remyelination fails in their absence [31], [60], [61]. Our results suggest that inhibition of NgR facilitated third ventricle progenitor activation and cell mobilization in response to demyelinated optic chiasm. It is also well documented that developmental origin of myelinating cells in the optic chiasm is an area in third ventricle neurogenic zone [62].

It has been also reported that inhibition of LINGO-1, a NgR co-receptor enhances oligodendrocyte differentiation and myelination in LINGO-/- [63] and in EAE model in LINGO-/- mice [64]. The authors of these reports, however, did not study expression of NgR in oligodendrocyte lineage cells, but did highlight the expression and role of LINGO-1 on neurons and mature oligodendrocytes. They showed that expression of LINGO-1, similar to its co-receptor NgR on neurons, can inhibit axonal regeneration by activation of RhoA signaling pathway; though LINGO-1 function on oligodendrocyte was not mediated through binding to NgR [65]–[67]. Huang et al. (2012); however, suggested that antagonizing NgR *in vitro* increased the number of PDGFRα+ OPCs and prolonged their processes but hampered their differentiation. They suggested that the effect of myelin inhibitory factors on OPC differentiation and process extending via NgR expressed by OPCs were mainly mediated via Erk1/2 and PI3/Akt signaling pathways and also involved various cell proliferation and differentiation effects [68], [69], including oligodendrocyte differentiation [17].

In the present study, we observed a reduced demyelination area at 7 dpi in the optic chiasm of siNgR treated groups. Two major conclusions can be drawn from our observations. First, inhibition of NgR could induce a neuroprotective effect against demyelination. Second, NgR signaling exerts a negative role on progenitor cell migration and myelin repair. Yu and coworkers suggested that in addition to stimulating axon regeneration, NgR inhibition might also be neuroprotective, contributing to the overall functional recovery after spinal cord injury, which can be attributed to neuroprotective effect of NgR inhibition. However, the mechanism underlying such possible effects remains to be investigated.

To our knowledge, the current data are the first to show that the therapeutic effect of NgR inhibition on functional myelin recovery in a non-immune model of demyelination. Further studies need to be carried out to elucidate the molecular mechanisms by which NgR exerts its inhibitory effects on plasticity *in vivo*. Such information about the nature of the changes in the endogenous cell niche in response to demyelination will clarify to what extent the potential of self-repair in adult CNS can be enhanced following damage. Such research would contribute to therapeutic perspectives for successful repair in demyelinating disorders including Multiple Sclerosis.

## Supporting Information

Figure S1 **Third ventricle response to LPC-induced demyelination.** (A–C) Double immunostaining of BrdU (red) and Olig2, GFAP and PSA-NCAM (green)-labeled cells in coronal section of third ventricle in saline treated animals showed that progenitor cells residing in third ventricle wall were not mitotically active. (D–F) Brdu (red)/Olig2 (green) double staining in third ventricle of LPC treated animals at 3, 7 and 14 dpi respectively. The number of BrdU+/Olig2+ cells increased at 7 dpi (E) compared to control. (G–I) The number of BrdU (red) and PSA-NCAM (green) positive cells in the third ventricle of LPC treated animals at 3(G), 7 (H) and 14 dpi (I) respectively. There were no remarkable changes between these groups.
(PDF)

Table S1 **Four different sequences of siRNA against NgR were combined in the same tube for injection.**
(PDF)

Table S2 **Primary antibodies used in this study.**
(PDF)

Table S3 **Secondary antibodies used in this study.**
(PDF)

Raw Data S1 **Raw data are presented as an attached Excel file.**
(XLSX)
