# Supplementary material for: Nogo Receptor Inhibition Enhances Functional Recovery following Lysolecithin-Induced Demyelination in Mouse Optic Chiasm
Source: PLoS One. 2014 Sep 3;9(9):e106378. doi: 10.1371/journal.pone.0106378 (PMC4153612; doi:10.1371/journal.pone.0106378)
Supplement: Figure S1 — Third ventricle response to LPC-induced demyelination. (A–C) Double immunostaining of BrdU (red) and Olig2, GFAP and PSA-NCAM (green)-labeled cells in coronal section of third ventricle in saline treated animals showed that progenitor cells residing in third ventricle wall were not mitotically active. (D–F) Brdu (red)/Olig2 (green) double staining in third ventricle of LPC treated animals at 3, 7 and 14 dpi respectively. The number of BrdU+/Olig2+ cells increased at 7 dpi (E) compared to control. (G–I) The number of BrdU (red) and PSA-NCAM (green) positive cells in the third ventricle of LPC treated animals at 3(G), 7 (H) and 14 dpi (I) respectively. There were no remarkable changes between these groups. (PDF) [file pone.0106378.s001.pdf]

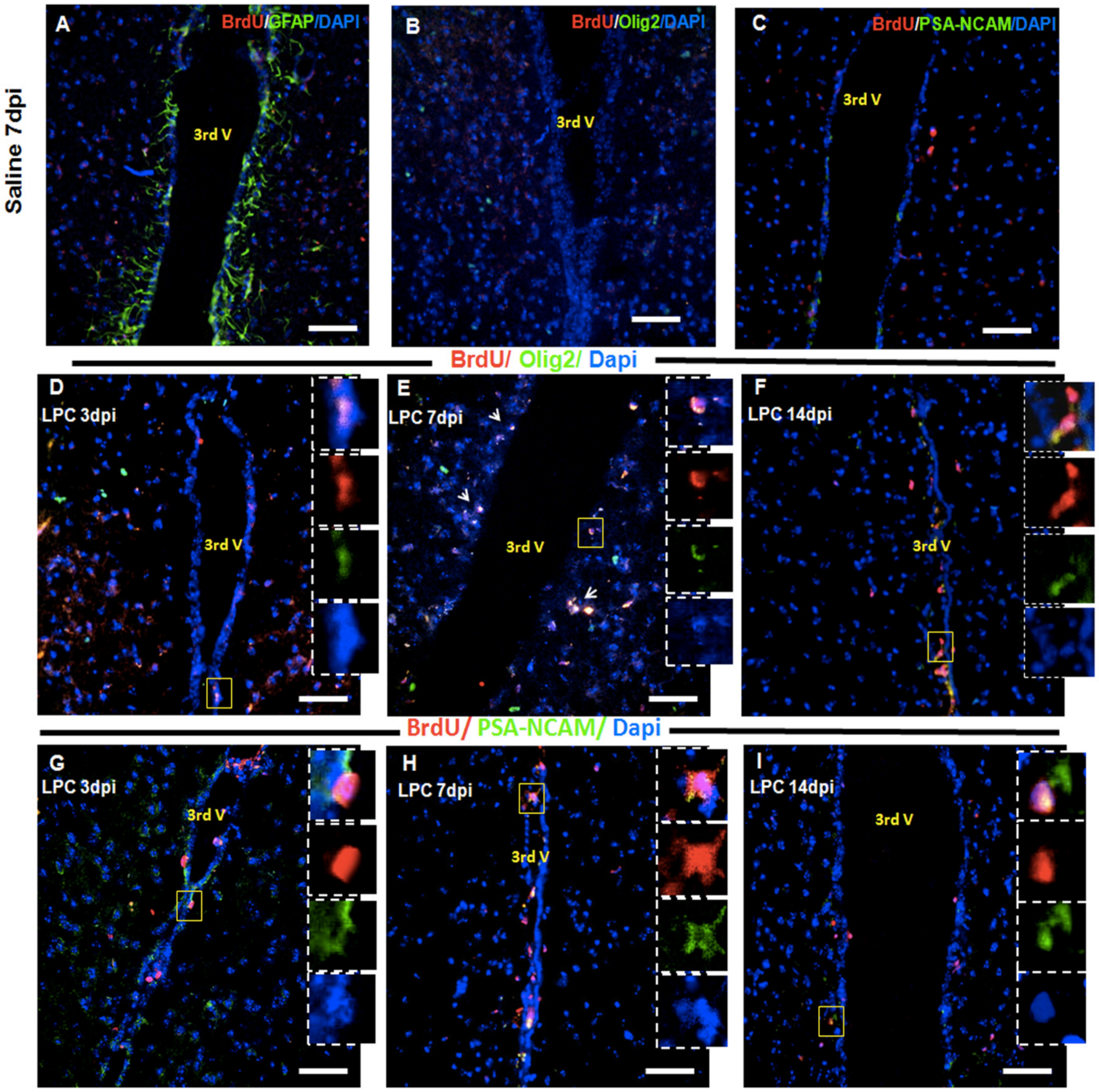

Figure S1. Third ventricle response to LPC-induced demyelination. (A-C) Double immunostaining of BrdU (red) and Olig2, GFAP and PSA-NCAM (green)-labeled cells in coronal section of third ventricle in saline treated animals showed that progenitor cells residing in third ventricle wall were not mitotically active. (D-F) Brdu (red)/Olig2 (green) double staining in third ventricle of LPC treated animals at 3, 7 and 14 dpi respectively. The number of BrdU+/Olig2+ cells increased at 7 dpi (E) compared to control. (G-I) The number of BrdU (red) and PSA-NCAM (green) positive cells in the third ventricle of LPC treated animals at 3(G), 7 (H) and 14 dpi (I) respectively. There were no remarkable changes between these groups.
