# Supplementary material for: Nogo Receptor Inhibition Enhances Functional Recovery following Lysolecithin-Induced Demyelination in Mouse Optic Chiasm
Source: PLoS One. 2014 Sep 3;9(9):e106378. doi: 10.1371/journal.pone.0106378 (PMC4153612; doi:10.1371/journal.pone.0106378)
Supplement: Table S1 — Four different sequences of siRNA against NgR were combined in the same tube for injection. (PDF) [file pone.0106378.s002.pdf]

Table S1. Four different sequences of siRNA against NgR were combined in the same tube for injection.

siRNA	Sequence (5'to 3')	Sense sequence (5' to 3')
**siRNA A**	CACCCTCTGGATCTACTCCAA	CCCUCUGGAUCUACUCCAATT
**siRNA B**	CACCCTCTTTCTCTTCAACAA	CCCUCUUUCUCUUCAACAATT
**siRNA C**	AAGGAGCAGGACTCAGAACAA	GGAGCAGGACUCAGAACAATT
**siRNA D**	CTGGTGGATTATAAGCCCAAA	GGUGGAUUAUAAGCCCAAATT

Selected sequences were subjected to a BLAST search (www.ncbi.nlm.nih.gov PubMed) to ensure that there was no significant homology with other genes. NgR-siRNA Cat no: SI02722888, SI02699186, SI02748333, SI02678249) were purchased from Qiagen.
