# Supplementary material for: Nogo Receptor Inhibition Enhances Functional Recovery following Lysolecithin-Induced Demyelination in Mouse Optic Chiasm
Source: PLoS One. 2014 Sep 3;9(9):e106378. doi: 10.1371/journal.pone.0106378 (PMC4153612; doi:10.1371/journal.pone.0106378)
Supplement: Table S2 — Primary antibodies used in this study. (PDF) [file pone.0106378.s003.pdf]

Table S2. Primary antibodies used in this study.

Primary Antibody	Catalog No.	Isotype	Host	Dilution for IHC	Manufacture
BrdU	MCA2060	IgG2a	Rat	1/500	abD Serotec
Olig2	AB9610	IgG	Rabbit	1/300	Millipore\
GFAP	Z0334	IgG	Rabbit	1/500	Dako
PSA-NCAM	AbC0019	IgM	Mouse	1/400	AbCys
MOG	MAB5680	IgG1	Mouse	1/100	Millipore
NF200	N4142	IgG	Rabbit	1/200	Sigma
Iba1	Ab15690	IgG2b	Mouse	1/50	Abcam
