# Supplementary material for: Nogo Receptor Inhibition Enhances Functional Recovery following Lysolecithin-Induced Demyelination in Mouse Optic Chiasm
Source: PLoS One. 2014 Sep 3;9(9):e106378. doi: 10.1371/journal.pone.0106378 (PMC4153612; doi:10.1371/journal.pone.0106378)
Supplement: Table S3 — Secondary antibodies used in this study. (PDF) [file pone.0106378.s004.pdf]

Table S3. Secondary antibodies used in this study.

SecondryAntibody	Catalog No.	Isotype	Host	Anti	Dilution for IHC	Company
Alexa Fluor 594	A11007	IgG(H+L)	Goat	Rat	1/500	Invitrogen
Alexa Fluor 488	A-11030	IgG(H+L)	Goat	Rabbit	1/500	Invitrogen
Alexa Fluor 488	A21042	IgM	Goat	Mouse	1/500	Invitrogen
Alexa Fluor 488	A-11001	IgG(H+L)	Goat	Mouse	1/500	Invitrogen
Alexa Fluor 488	A-21141	IgG2b	Goat	Mouse	1/500	Life Technologies
Alexa Fluor 555	A-31572	IgG(H+L)	Donkey	Rabbit	1/500	Invitrogen
